# Effect of cryopreservation and semen extender on extracellular vesicles isolated from bull semen

**DOI:** 10.3389/fvets.2024.1437410

**Published:** 2024-07-30

**Authors:** Emanuele Capra, Roberto Frigerio, Barbara Lazzari, Federica Turri, Giulia Gaspari, Luisa Pascucci, Alessandra Stella, Anna Lange Consiglio, Flavia Pizzi, Marina Cretich

**Affiliations:** ^1^Institute of Agricultural Biology and Biotechnology (IBBA), National Research Council (CNR), Lodi, Italy; ^2^Institute of Chemical Sciences and Technologies “Giulio Natta” (SCITEC), National Research Council (CNR), Milan, Italy; ^3^Department of Veterinary Medicine and Animal Science (DIVAS), University of Milan, Lodi, Italy; ^4^Department of Veterinary Medicine, University of Perugia, Perugia, Italy

**Keywords:** extracellular vesicles, seminal plasma, extender, cryopreservation, miRNA, bull

## Abstract

**Introduction:**

Semen cryopreservation is the most popular practice for semen production for artificial insemination and *in vitro* fertilization in cattle. The Seminal plasma contains extracellular vesicles (spEVs) which modulate sperm viability and function during oocyte fecundation. The study of spEVs in frozen-thawed semen doses may yield novel indicators for predicting bull fertility, but the presence of the semen extender may hinder molecular profiling of spEVs. The aim of this study was to provide extensive characterization of EVs isolated from seminal plasma before and after the cryopreservation process and the addition of a commercial animal protein-free semen extender to understand the potential influence of EVs originating from the extender in hindering the use of spEVs derived biomarkers for assessment of bull fertility.

**Methods:**

EVs were isolated from the seminal plasma (with or without the extender), from the cryopreserved straw devoid of spermatozoa, and from the extender using two different methods, ultracentrifugation (UC) and size exclusion chromatography (SEC), and characterized for their structure and composition.

**Results:**

Physical characterization of EVs showed that size and particle numbers were related to the method of isolation. spEVs were larger but less abundant (UC: 168.9 nm, *n* = 2.68 × 10^9^; SEC: 197.0 nm, *n* = 6.42 × 10^9^) compared to extender EVs (UC: 129.0 nm, *n* = 2.68 × 10^11^; SEC: 161.8 nm, *n* = 6.47 × 10^11^). Western blotting analysis (WB) confirmed the presence of typical EV markers in spEVS: the membrane bound CD9 (25 kDa) and the luminal markers Alix (96 kDa) and TSG101 (48 KDa). Although Transmission Electron Microscopy confirmed the presence of a lipid bilayer structure in all preparations, no specific EV markers were detected in the vesicles isolated from extender when the Single Molecule Array (SiMoa) was used. A total of 724 Bos taurus miRNAs were identified in at least one preparation. The percentage of miRNAs identified in EVs from the extender (0.05%−0.49% of the total reads) was lower than in the preparation containing spEVs (10.56%–63.69% of the total reads). Edge-R identified a total of 111 DE-miRNAs between EVs isolated from the extender by two methods. Among them, 11 DE-miRNAs (bta-miR-11980, bta-miR-11987, bta-miR-12057, bta-miR-1246, bta-miR-125b, bta-miR-181b, bta-miR-2340, bta-miR-2358, bta-miR-2478, bta-miR-2898, and bta-miR-345-3p) were also abundant in EVs isolated from seminal plasma preparations with extender.

**Conclusion:**

This study clearly demonstrates that the presence of the extender does not prevent the characterization of spEVs in cryopreserved semen. However, the molecular profiling of spEVs can be influenced by the isolation method used and by the presence of some miRNAs from the extender. Therefore, in such studies, it is advisable to characterize both spEVs and the vesicles isolated from the extender.

## 1 Introduction

In cattle farming, male infertility is closely related to production efficiency and is a major cause of economic losses. Bulls have a high inter-individual variability in fertility, hence assessment of sperm quality and sire fertility rate is important for breeding plans (1). In some cases, despite the use of bulls with high genetic value, full-term pregnancies are not obtained (2). This is also the case when employing high-merit bulls, based on their spermatozoa motility and morphology (3, 4), since this characteristic doesn't necessarily indicate the absence of molecular defects of the spermatozoa, which might affect fertilization or contribute to abnormal embryo development (5).

To optimize reproductive success many cattle breeders make use of assisted reproductive technologies such as sperm cryopreservation, artificial insemination (AI) and *in vitro* fertilization (IVF). AI strategies vary around the world. Far Eastern and some European countries favor the use of fresh semen, but the use of cryopreserved semen remains the most popular practice worldwide (6).

The use of cryopreserved semen allows breeders to estimate the bull potential fertility rate, by the assessment of specific events such as non-return rate (NRR) or estimated relative conception rate (ERCR). Several advanced technologies can be used to examine the quality of spermatozoa, such as computer-assisted semen analysis (CASA) and flow cytometry (FCM), which can provide accurate and objective evaluation of sperm function. The combination of kinetic semen parameters originating from CASA and DNA analysis based on FCM seems able to classify fertility levels in bulls even in the high-fertility range (7). Moreover, to better predict bull fertility, a combined approach that integrates semen quality evaluation with advanced technologies and sperm molecular characterization using different modern approaches can be advantageous (8). In recent years, several studies combining methods traditionally used in spermatology with techniques of molecular biology that target sperm microRNAs (miRNAs) (9) or integrating multi-omics approaches like transcriptomic, proteomic and metabolomic analysis (10), were able to identify molecules potentially influencing bull fertility.

Although some specific molecular markers were found to be associated with different sperm quality traits, like sperm motility, acrosome, plasma membrane and DNA integrity, their potential for large-scale employment is still limited (11).

These studies strictly evaluated molecular composition of sperm, but it is known that several other molecules present in the ejaculate can support oocyte fertilization and enhance embryo development.

Seminal plasma (SP) plays a key role in modulating fertility by modulating sperm viability and function, interacting with the various compartments of the female genital tract and serving as a carrier of signals regulating the female immune system (12, 13).

SP is a heterogeneous composite fluid which contains inorganic ions, specific hormones, proteins, cholesterol and extracellular vesicles that interact with the various compartments of the tubular genital tract preparing for an eventual successful pregnancy (13). Extracellular vesicles (EVs) are membrane-enclosed microparticles originating from outward budding of plasma membranes by active secretion that that mediate cell to cell communication in proximity to, or distant from, the cells of origin. EVs are usually isolated from complex body fluids by different methods such as ultracentrifugation, density gradient centrifugation, precipitation with polymers and size exclusion chromatography (14).

Seminal plasma contains a very high number of EVs compared to other biological fluid (15). EVs contained in the seminal plasma (spEVs) are released by the testis, epididymis and male accessory glands, such as prostate and vesicular glands (16). Accordingly, spEVs exchange active molecules with mature sperm and endometrial epithelial cells and regulate sperm motility, capacitation, and acrosome reaction and also facilitate the safe transit of spermatozoa through the female genital tract (17). Supplementation of spEVs was observed to improve fertilizing capacity of bulls in *in-vitro* experiments (18). Alteration of SP and spEV molecular composition was also reported in different andrological diseases. For example, proteomic characterization of seminal plasma collected from infertile men with unilateral varicocele showed dysregulation of the exosome associated protein (19), while miRNA profiles of spEVs in human patients with oligoasthenozoospermia was altered. In cattle, different studies reported potential seminal plasma biomarkers for bull fertility. Metabolomic characterization of SP identifies specific metabolites whose abundance potentially correlates with bull fertility (20). Recently, seminal plasma small RNA profiling in high- and low-fertile Holstein bulls revealed alteration in miRNAs targeting genes potentially regulating sperm function and structure, fertilization, and placental and embryo development (21).

However, all these studies reported molecular characterization of spEVs isolated from fresh ejaculate. In order to optimize production and preserve individuals of high genetic value, natural mating has been passed over to make way for AI, which involves cryopreservation of semen with extender. A preliminary study is needed to clarify whether the vesicular component present in the extender can influence the detection of fertility markers in EVs.

This study aimed to characterize EVs isolated from seminal plasma before and after extender addition and cryopreservation, to evaluate the potential use of spEV-derived biomarkers from cryopreserved semen straws in assessing bull fertility.

## 2 Materials and methods

### 2.1 Isolation of EVs

#### 2.1.1 Biosample collection and semen quality

**Figure 1:**
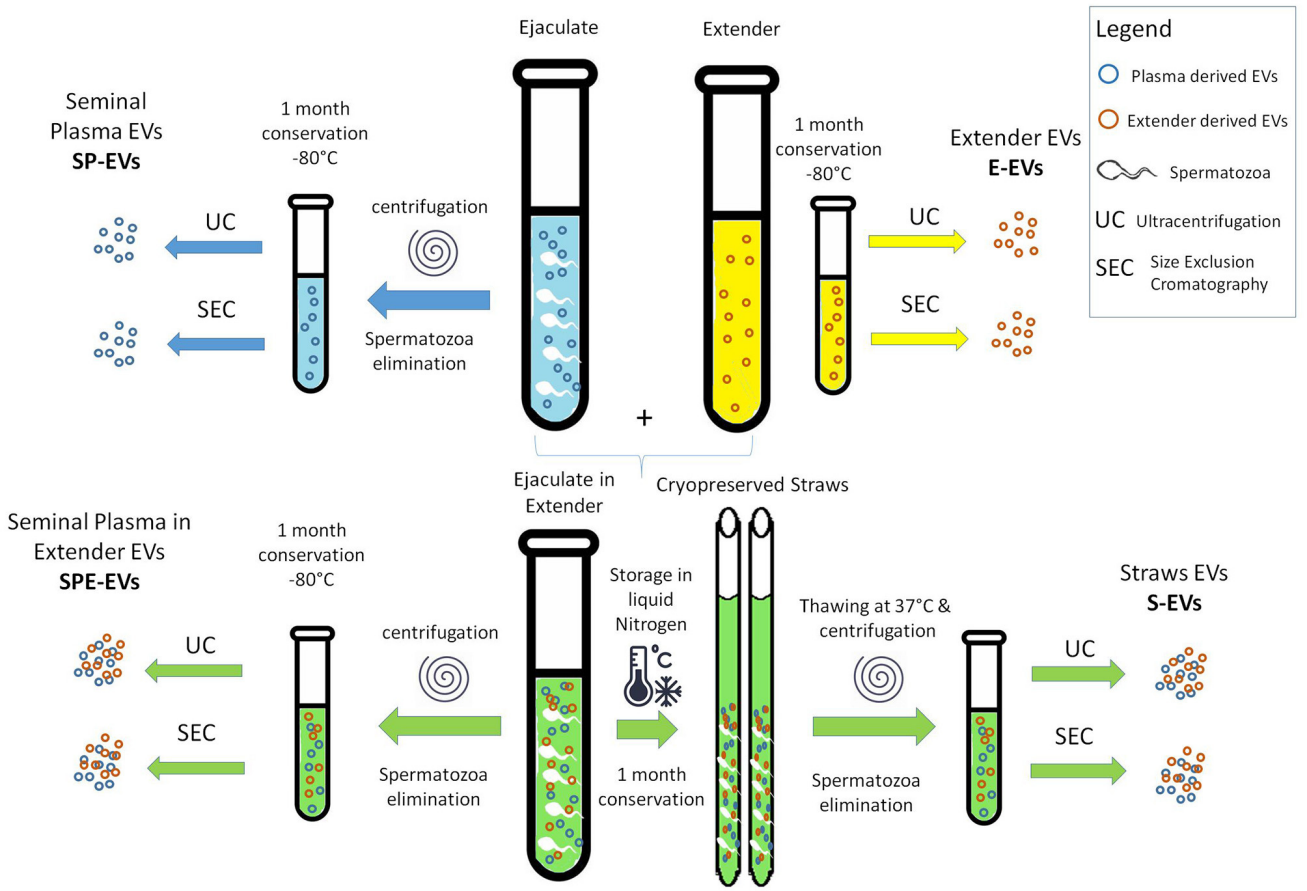
Scheme of the experimental design.

The experimental plan is reported in Figure 1. Five fresh ejaculates from five proven and fertile Italian Holstein bulls were collected at an Artificial Insemination (AI) center (GB GENETICS COFA SRL, Cremona, Italy) using an artificial vagina (Supplementary Table S1). Semen evaluation was performed by the personnel of the AI center on fresh samples. In particular semen concentration was evaluated with a Accucell photometer (IMV Technologies) and sperm motility was evaluated subjectively using a phase contrast microscope. Total motility and sperm kinetics parameters were assessed on post-thaw semen by CASA system (ISAS^®^v1, Proiser, R+D S.L., Paterna, Spain) combined with a phase contrast microscope (Nikon Optiphot) equipped with a negative phase contrast 10 × objective and integrated warmer stage and connected to a video camera (Proiser 782M, Proiser R+D).

#### 2.1.2 EV preparation

EVs were isolated as follows. Immediately after semen collection each ejaculate was divided (i) half was centrifuged (6,000 g for 5 min) to remove spermatozoa, and seminal plasma and then stored –at 80°C (SP). A second half was extended with a commercial animal protein-free semen extender (BioXcell: IMV, L'Aigle, France) and (ii) devoided of spermatozoa (6,000 g for 5 min) and the seminal plasma with extender stored at −80°C (SPE), or (iii) used to prepare straws for cryopreservation in liquid nitrogen (S). (iv) An aliquot of pure semen extender was also stored at −80°C (E). After 1 month, the cryopreserved straws (S) were thawed at 37°C for 1 min and centrifuged (6,000 g for 5 min) to remove the spermatozoa. SP, SPE, and E samples stored at −80°C were also thawed.

A pool of five bull's samples, was created from each (SP, SPE, S, and E) sample five that was split into three aliquots to obtain three biological replicates (*n* = 3). Samples underwent two sequential centrifugations at 4°C (600 g for 20′ and 4,000 g × 20′), and the supernatants were used for EV isolation by ultracentrifugation (UC) and size exclusion chromatography (SEC) to obtain the different preparations (SP-EVs_UC, SP-EVs_SEC, SPE-EVs_UC, SPE-EVs_SEC, S-EVs_UC, S-EVs_SEC, E-EVs_UC, and E-EVs_SEC) as reported in Table 1.

**Table 1:** The table reports the extracellular vesicle (EV) preparations obtained from different sources using two isolation methods: ultracentrifugation (UC) and size exclusion chromatography (SEC).

**Source**	**Replicates**	**EVs isolation method**	**EVs preparation name**
Seminal plasma	Pool 1	UC	SP-EVs_UC_1
Seminal plasma	Pool 2	UC	SP-EVs_UC_2
Seminal plasma	Pool 3	UC	SP-EVs_UC_3
Seminal plasma	Pool 1	SEC	SP-EVs_SEC_1
Seminal plasma	Pool 2	SEC	SP-EVs_SEC_2
Seminal plasma	Pool 3	SEC	SP-EVs_SEC_3
Seminal plasma in extender	Pool 1	UC	SPE-EVs_UC_1
Seminal plasma in extender	Pool 2	UC	SPE-EVs_UC_2
Seminal plasma in extender	Pool 3	UC	SPE-EVs_UC_3
Seminal plasma in extender	Pool 1	SEC	SPE-EVs_SEC_1
Seminal plasma in extender	Pool 2	SEC	SPE-EVs_SEC_2
Seminal plasma in extender	Pool 3	SEC	SPE-EVs_SEC_3
Cryopreserved straws	Pool 1	UC	S-EVs_UC_1
Cryopreserved straws	Pool 2	UC	S-EVs_UC_2
Cryopreserved straws	Pool 3	UC	S-EVs_UC_3
Cryopreserved straws	Pool 1	SEC	S-EVs_SEC_1
Cryopreserved straws	Pool 2	SEC	S-EVs_SEC_2
Cryopreserved straws	Pool 3	SEC	S-EVs_SEC_3
Extender	Pool 1	UC	E-EVs_UC_1
Extender	Pool 2	UC	E-EVs_UC_2
Extender	Pool 3	UC	E-EVs_UC_3
Extender	Pool 1	SEC	E-EVs_SEC_1
Extender	Pool 2	SEC	E-EVs_SEC_2
Extender	Pool 3	SEC	E-EVs_SEC_3

Ultracentrifugation was performed at 100,000 g (Beckman Coulter OptimaX, Milan, Italy), at 4°C for 1 h. The pellet was resuspended in a serum-free medium with 1% dimethylsulfoxide and stored at −20°C. SEC was performed on pEV10 column (IZON, Medford, MA, USA), following the manufacturer's instruction.

### 2.2 EV characterization

EVs isolated from each preparation were characterized according to the MISEV2023 guidelines (22).

#### 2.2.1 EVs nanoparticle tracking analysis

Number, dimension and quantity of isolated particles were determined by Nanoparticle tracking analysis (NTA) using a NanoSight NS300 system (Malvern Technologies, Malvern, UK) configured with 532 nm laser, according to the manufacturer's instruction and previously reported method (23). Each EV preparation was diluted to achieve a final volume of 1 ml in filtered PBS to obtain the ideal particle per frame (about 20–100 particles/frame). Samples were injected with a constant flow and three videos of 60 s were captured and analyzed with Malvern NTA software version 3.2. Particle size and concentration were expressed in nanometer (nm) and in particles/mL.

#### 2.2.2 Western blotting

EV proteins were evaluated by western blotting, 8 uL of reduction buffer (Laemmli buffer) was added to 32 uL of each EV preparation and the sample boiled for 5 min at 95°C; western blot analysis was performed according to method in previously published paper (24). All preparations were separated by SDS-PAGE (4%−20%, Mini-Protean TGX Precast protein gel, Bio-Rad) and blotted onto a nitrocellulose membrane (BioRad, Trans-Blot Turbo). To saturate non-specific sites a blocking step for 1 h with 5% (w/v) BSA in T-TBS (tris-buffered saline: 150 mM NaCl, 20 mM TrisHCl, pH 7.4, and 0.5% Tween 20) was performed. Primary antibody incubation with anti-CD9 (1:1,000, BD Pharmingen), anti-Alix (1:1,000, Santa Cruz, CA, USA), and anti-TSG101 (1:1,000, Novus Bio, Centennial, CO, USA) was performed overnight at 4°C. The next day membranes were washed three times with T-TBS, and incubated with the secondary antibodies horseradish peroxidase-conjugated (Jackson ImmunoResearch, Tucker, GA, USA) diluted 1:3,000 for 1 h. After final washing, the Bio-Rad Clarity Western ECL Substrate (Bio-Rad) was added, and signal detected using a Chemidoc XRS + (BioRad).

#### 2.2.3 Single molecule array

SiMoA beads conjugation was performed according to Quanterix Homebrew kit instructions using the recommended buffers (25). In pan-tetraspanin three-step assay, beads solution was prepared at the concentration of 2 × 10^7^ beads/ml in Bead Diluent. The detector antibody (biotinylated CD9, CD63, and CD81 antibodies by Ancell) solutions (0.3 μg/ml) were diluted in Homebrew Sample Diluent (Quanterix), while the EV preparations were diluted 1:4 in Homebrew Sample Diluent (Quanterix). Twenty five μl of beads were transferred into a 96 microwell plate and 100 μl of diluted sample added, incubated for 30 min at 25°C at 800 rpm. After incubation, beads were washed with an automatic plate-washer and then incubated for 10 min with 100 μl of detector antibody. Then, beads were washed and incubated for 10 min with a 150 pM SBG solution (in SBG Diluent, Quanterix). After SBG incubation step, final washes were performed and the plate was inserted into the Quanterix SR-X instrument for analysis where RGP is automatically added. Data were analyzed and processed by Reader Software SiMoA 1.1.0.

#### 2.2.4 Transmission electron microscopy

For EV analysis, 10 μl of each vesicle suspension were placed on Parafilm (Bemis, Neenah, WI, USA). Formvar-coated copper grids (Electron Microscopy Sciences, Hatfield, PA, USA) were placed on top of the drops with the coated side facing the suspension. The EVs were adsorbed onto the grid for 1 h at room temperature in a humidity chamber. The grids were then briefly washed in 0.1 M phosphate buffer saline (PBS), pH 7.3, rinsed with distilled water and contrasted with 2% uranyl acetate (Electron Microscopy Sciences). The grids were observed under a Philips EM 208 microscope equipped with a digital camera (University Centre for Electron and Fluorescence Microscopy, CUMEF, Perugia, Italy).

### 2.3 miRNA profiling of EVs

#### 2.3.1 RNA isolation

RNA was extracted from all isolated EVs with TRIzol (Invitrogen, Carlsbad, CA, USA), following the manufacturer's instructions. After centrifugation (12,000 g 15′, 4°C), upper aqueous solution containing RNA was cleaned-up with the NucleoSpin miRNA kit (Macherey–Nagel, Germany), following the protocol in combination with TRIzol lysis with small and large RNA in one fraction (total RNA). Concentration and quality of RNA were determined using RNA 6000 Pico Kit for 2100 Bioanalyzer (Santa Clara, CA, USA). The isolated RNAs were stored at −80°C until use.

#### 2.3.2 Library preparation and sequencing

In total, 24 small RNA libraries were obtained from EVs isolated by different methods (*n* = 2), different preparations (*n* = 4), and replications (*n* = 3). Small RNA libraries were prepared using QIAseq miRNA Library Kit (Qiagen, Hilden, Germany), according to the manufacturer's instructions. Concentration and profile of libraries were determined by High Sensitivity DNA kit for Agilent 2100 Bioanalyzer. Libraries were pooled and sequenced on a single lane of Illumina Novaseq X (San Diego, CA, USA).

#### 2.3.3 Data analysis

Illumina raw sequence analysis was carried out with the nf-core/smrnaseq pipeline, v2.2.4 (26). The pipeline performs a number of steps, encompassing sequence quality control and trimming, and reads alignment to *Bos taurus* miRNAs available at miRBase (http://www.mirbase.org/). MiRNA identification and quantification is performed by MiRDeep2 and its modules. The Bioconductor edgeR package (version 2.4) was used to identify statistically significant differential expression between groups of samples (false discovery rate [FDR] < 0.05) (27). MicroRNA cluster analysis was performed with Genesis (version1.8.1) (28). Venn diagrams were produced with InteractiVenn (29). Statistics of SiMoA data was performed by GraphPad PRISM 9.0 (La Jolla, California); *t*-test analysis was used to evaluate the significant different, *p*-value < 0.05 was considered significant.

## 3 Results

### 3.1 Biosamples collection, semen quality and isolation of EVs

All the data about semen quality are available in the Supplementary Table S2.

Semen volume was between 3.16 ml and 5.14 ml, and sperm concentration ranged from 575 to 1,188 × 106 sperm/ml. The subjective motility evaluated on fresh semen at the AI center was between 77% and 80%. On post-thaw samples the motility evaluated by CASA system ranged from 65.3% and 77.8%.

EVs were collected from different preparations (Figure 1):

- EVs from seminal plasma devoid of spermatozoa (SP-EVs).
- EVs from seminal plasma devoid of spermatozoa, with addition of the extender (SPE-EVs).
- EVs from cryopreserved sperm, devoid of spermatozoa (S-EVs).
- EVs from the extender (E-EVs).

All EV preparations were obtained in triplicate by combining a pool of seminal plasma from five different bulls, using two different isolation methods: Ultracentrifugation (UC) and Size Exclusion Chromatography (SEC). Table 1 reports all EV preparations analyzed in this study.

### 3.2 EV characterization

**Figure 2:**
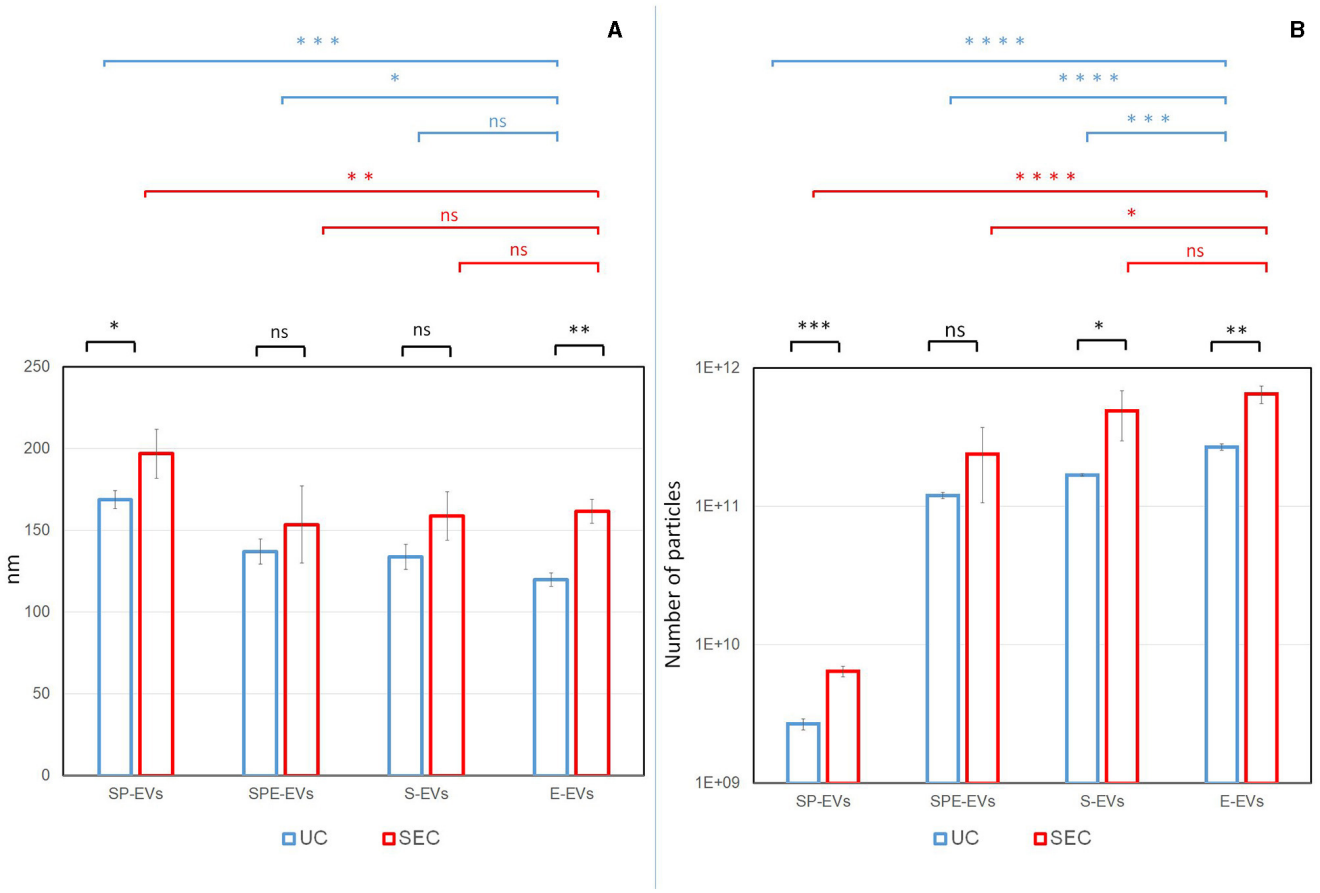
Particle size distribution **(A)** and concentration **(B)** were assessed by Nanoparticle Tracking Analysis (NTA). For each preparation (SP-EVs: EVs from seminal plasma devoid of spermatozoa, SPE-EVs: EVs from seminal plasma devoid of spermatozoa with addition of the extender, S-EVs: EVs from cryopreserved sperm devoid of spermatozoa, and E-EVs: EVs from the extender) and isolation methods (UC: Ultracentrifugation, SEC: Size Exclusion Chromatography), the replicates average for size and particle number were reported. Statistical differences were calculated: between two methods (in black), between different separation for UC (in blue) and between different separation for SEC (in red). *P-*value: (*) < 0.05, (**) < 0.01, (***) < 0.001, (****) < 0.0001.

EVs isolated from each preparation were characterized according to the MISEV2023 guidelines (22). In order to evaluate the size distribution and the particle concentration, Nanoparticle Tracking Analysis (NTA) was performed on each sample. Overall, some statistically significant differences in terms of EV recovery yield and size comparing UC and SEC isolation methods and different preparations, were noted (Figure 2). A significantly higher number of larger particles were recovered from the SEC preparations SP-EVs (SEC: 197.0 nm ± 14.9; UC: 168.9 nm ± 7.6) and E-EVs (SEC: 161.8 nm ± 7.3; UC: 120.0 nm ± 4.2), except for SPE-EVs (SEC: 153.7 nm ± 23.5; UC: 137.2 nm ± 7.6), and S-EVs (SEC: 158.8 nm ± 16.7; UC: 133.8 nm ± 7.8) for which differences in size were not significant, compared to UC preparations (Figure 2A).

For both isolation methods, preparations containing the extender: SPE-EVs (SEC, *n* = 2.38 × 10^11^ nm; UC, *n* = 1.20 × 10^11^ nm), S-EVs (SEC, *n* = 4.88 × 10^11^ nm; UC, *n* = 1.69 × 10^11^ nm), and E-EVs (SEC, *n* = 6.47 × 10^11^ nm; UC, *n* = 2.68 × 10^11^ nm) showed a significantly higher number of particles when compared to SP-EVs (SEC, *n* = 6.42 × 10^9^ nm; UC, *n* = 2.68 × 10^9^ nm) (Figure 2B). In addition, E-EVs were significantly smaller when compared to SP-EVs and affected the size distribution of SPE-EVs and S-EVs (Figure 2A).

It is worth noting that NTA particle count is not specific for EVs and that protein aggregates, lipoproteins and other non-vesicular material could contribute to the particle count providing an overestimation of EV concentration. These issues can be addressed by performing additional analyzes at the molecular level.

**Figure 3:**
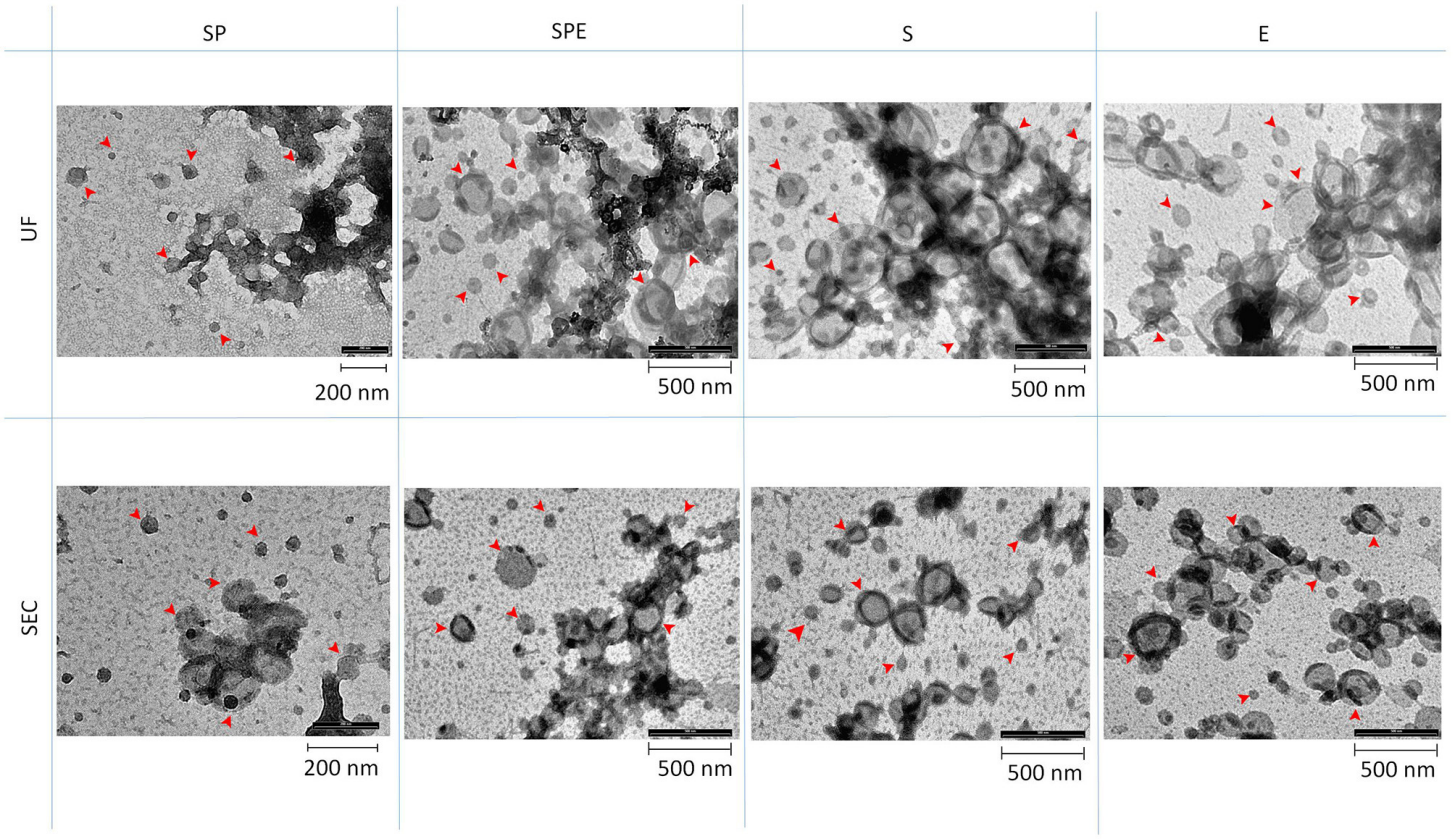
Transmission Electron Microscopy (TEM) for different preparations: SP, SPE, S, and E, isolated by Ultracentrifugation (UC) and Size Exclusion Chromatography (SEC). Arrows indicate EVs.

**Figure 4:**
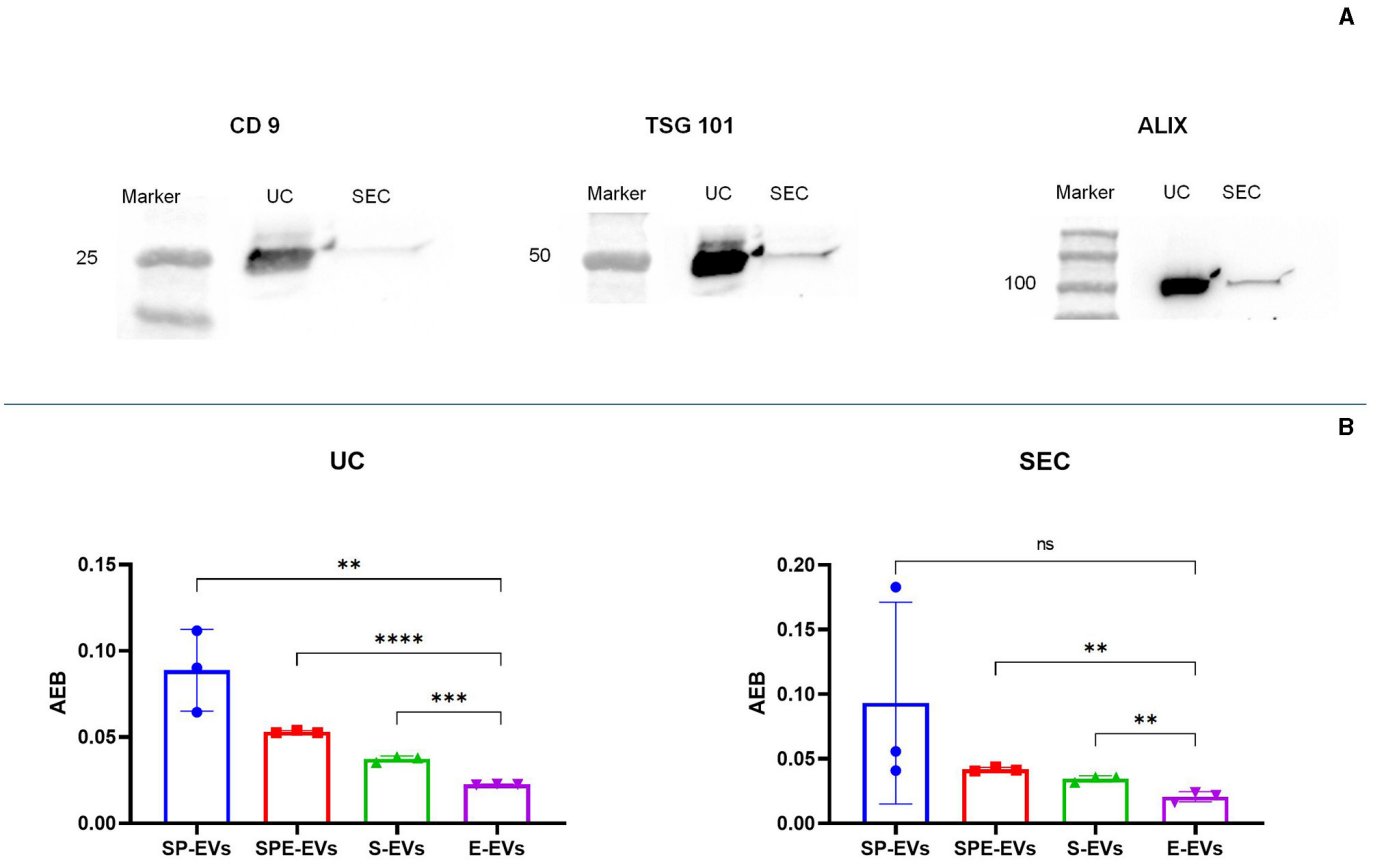
Characterization of vesicular protein markers using: **(A)** western blot characterization of sp-EV preparation using a membrane marker (CD9) and internal markers (TSG 101 and Alix), **(B)** single molecule array (SiMoa) was performed on all preparations in order to evaluate tetraspanin expression; signal is reported in AEB (Avarege Enzyme per Bead), intensity is proportional at the amount of tetraspanin (CD9, CD63 or CCD81) expressed on EVs' surface; in this way AEB signal allowed us to quantify the amount of EVs in each sample. Statistical difference was calculated by *t*-test analysis, (p value < 0.05, significant). P-value: (^**^) < 0.01, (^***^) < 0.001, (^****^) < 0.0001.

Transmission electron microscopy (TEM) showed the presence of EVs in all preparations containing plasma and in the extender for both isolation methods (Figure 3). Western blotting analysis (WB) was performed on all SP-EV preparations isolated by UC and SEC, to confirm the presence of typical EVs' markers: the membrane bound CD9 (25 kDa) and the luminal markers Alix (96 kDa) and TSG101 (48 KDa) (Figure 4A). UC derived SP-EVs give rise to more intense signals, suggesting a higher recovery. A quantitative analysis of EVs in three different pools of each preparation was performed by Single Molecule Array (SiMoa), according to recently developed protocols for ultra-sensitive pan-tetraspanin detection (25) (Figure 4B). In this bead-based immune-assay, antibodies against tetraspanin (CD9, CD63, and CD81) conjugated onto paramagnetic beads are used to capture EVs, while detection is based on the use of biotinylated anti-tetraspanin antibodies. Exploiting SiMoA technology, we were able to detect tetraspanin signals that is directly proportional at the amount of EVs in each preparation. Signal is expressed in Average Enzyme per Bead (AEB) (Supplementary Table S3). Statistical significate was evaluated comparing all EVs samples with diluent preparation; it was observed that all UC preparations have a significantly different compare to extender, the same was observed for the SEC preparations but excluding SP-EVs that do not show a statistically different with E-EVs. Overall, a clearly trend is evident, SP-EVs gave a high signal, while SPD-EVs and S-EVs were less intensive but higher then E-EVs. The most striking result is the absence of tetraspanin signal in E-EVs (AEB signal is similar to the negative control).

### 3.3 miRNA profiling in EVs

**Figure 5:**
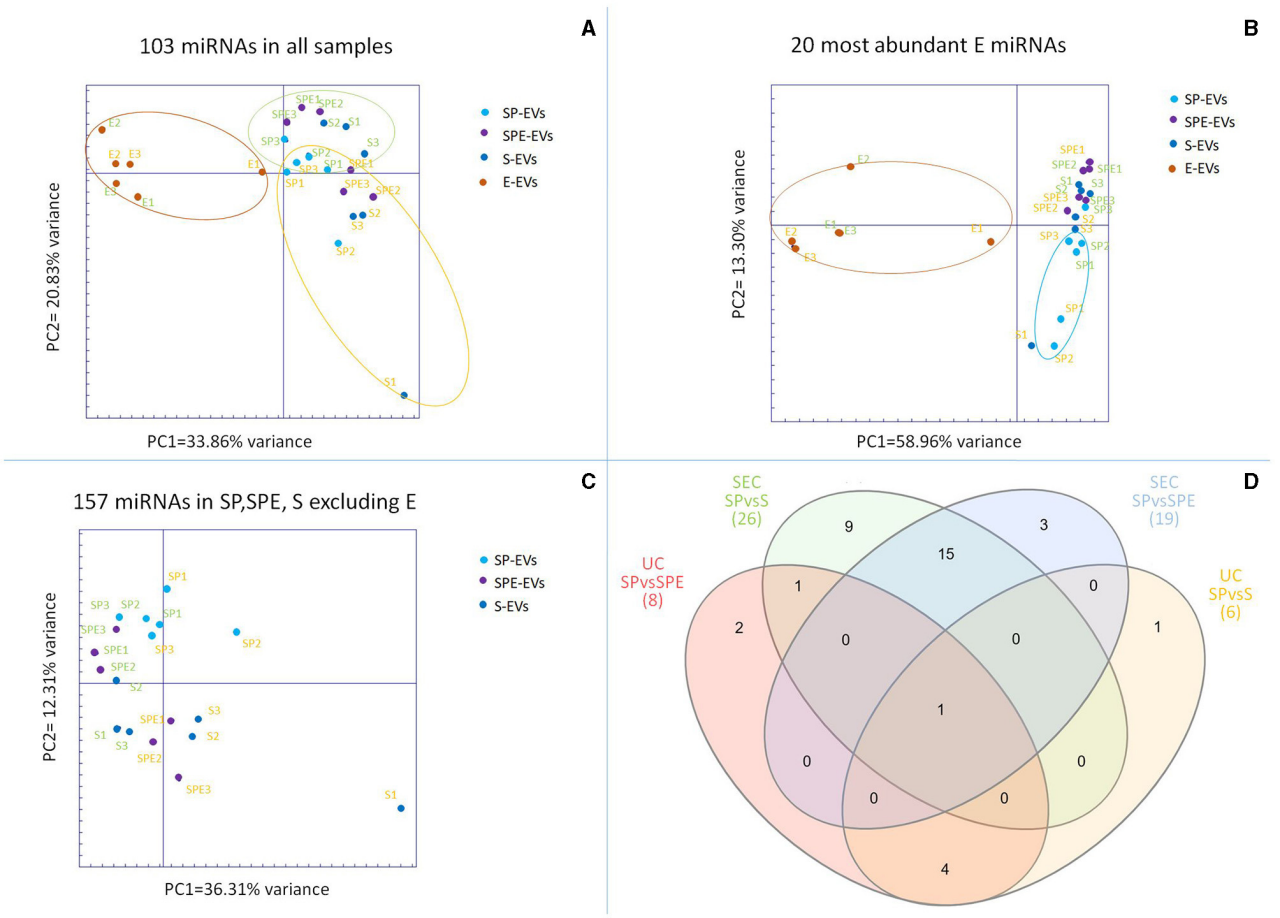
**(A)** Principal component analysis (PCA) of the 103 miRNAs present in all samples, **(B)** PCA for the 20 most abundant miRNAs in the extracellular vesicles (EVs) isolated from the extender (E-EVs), **(C)** PCA for miRNAs present in EVs isolated from seminal plasma preparations (SP-EVs, SPE-EVs, and S-EVs). In green EVs isolated by SEC and in orange by UC. **(D)** Venn diagram of shared differentially expressed miRNAs (DE-miRNAs) found in EVs across seminal plasma preparations, with (SPE and S) or without (SP) the extender using Ultracentrifugation (UC) and Size Exclusion Chromatography (SEC).

About 23.73 ± 12.49 million reads were sequenced for EVs isolated from all the preparations (Supplementary Table S4). The proportion of sequences classified as miRNAs was variable in the different datasets, depending on the type of preparation and isolation method. Overall, a greater proportion of miRNA sequences among total sequences was observed in datasets derived from the UC isolation method. In agreement with EV characterization, miRNA profiling showed a very low content of miRNAs in the E-EV samples (0.05% with UC, 0.10% with SEC), whereas the proportion of miRNAs in total sequences in other preparations was about 23.89%, with the exception of SP-EVs obtained by UC, showing the highest percentage of assigned miRNAs (59.31%). A total of 724 *Bos taurus* miRNAs was detected in at least one sample (Supplementary Table S5). Principal component analysis of the 103 miRNAs counted at least once in all 24 samples clearly separates E-EVs vs. other samples on Principal Component 1, explaining 33.86% of the variance, indicating that the expression of miRNAs in the extender was different from SP-EVs (Figure 5A). Seminal plasma EVs from different preparations (SP, SPE, and S) cluster closely and are not distinguishable, bur their miRNA content seems, in part, to be influenced by the methods used for vesicle isolation. Observing PCA results on the 20 most abundant miRNAs present in the extender, it is evident that EVs from the extender differ from those from seminal plasma, but it is also evident that 5 out of 6 SP samples group apart from samples from other two preparations (SPE and S) containing the extender (Figure 5B).

PCA of miRNAs detected in the three EV preparations containing seminal plasma, excluding the extender, showed a partial separation between EVs isolated with UC and SEC (Figure 5C).

The comparison between EVs from the extender and EVs from other preparations containing seminal plasma showed 48 and 94 differentially expressed miRNAs (DE-miRNAs) for UC and SEC isolation methods, respectively (Supplementary Table S3) of which 31 DE-miRNAs were identified for both isolation methods. DE-miRNAs calculated between SP, SPE and S groups in EVs isolated by both UC and SEC methods show no variations among preparations containing the extender (Supplementary Table S6). On the contrary, SP showed 8 (UC) and 19 (SEC) DE-miRNAs and 6 (UC) and 26 (SEC) DE-miRNAs when compared to SPE and S, respectively (Figure 5D). Considering, all the 111 DE-miRNAs (E-EVs vs. other, by UC or SEC) and 36 DE-miRNAs (SP-EVs vs. SPE-EVS or S-EVS, by UC and SEC) we found 11 DE-miRNAs (bta-miR-11980, bta-miR-11987, bta-miR-12057, bta-miR-1246, bta-miR-125b, bta-miR-181b, bta-miR-2340, bta-miR-2358, bta-miR-2478, bta-miR-2898, and bta-miR-345-3p) that were over-represented in the EVs isolated from extender and were found abundantly in spEVS with added extender.

## 4 Discussion

To the best of our knowledge this is the first work describing spEV characterization in fresh ejaculate and after semen extender addition and cryopreservation. For this study, EVs were isolated using the two gold standard methods, UC and SEC. When compared with the UC method, SEC showed an increase of separation yield and a different distribution of oversized particles. A previous work evaluating the impact of isolation methods on human spEV characterization using alternative methods based on precipitation reagents, showed that different EV isolation methods displayed different size profiles, including size mean and mode, when compared to the ones obtained with the UC method (30). Although we did not find any other studies reporting application of SEC and UC methods in isolating spEVs, both methods were applied for isolating EVs from plasma and cell culture conditioned medium, giving contrasting results. NanoFCM analyzes of plasma EVs showed that a higher number of particles with similar size were isolated by SEC compared to UC (31), but SEC isolated fewer EVs particles in conditioned medium (32). Interestingly, in agreement with our results, the plasma miRNAome differed between UC and SEC isolation methods (31).

In our work, there was a noticeable difference using NTA between EVs isolated from seminal plasma and extender for both isolation methods. spEVs were oversized particles but less abundant, while extender particles were smaller but found in a higher number. Preparations containing both seminal plasma and extender showed particles of an intermediate size and an EV abundance comparable with the samples containing extenders. NTA is a robust method, able to accurately measure the size distribution and the total concentration of the EV preparation being studied, but it is not specific for EVs, detecting also other particles, such as protein aggregates, lipoproteins and cellular debris (33). The extender BioXcell contains vegetable components, and its chemical composition reports the presence of soy lecithin (34). This substance is able to form vesicles with a size similar to that of the EVs collected from the extender (35). TEM analysis confirmed the presence of EVs in all preparations including the extender, conversely SIMOA analysis affirmed the presence of tetraspanin: CD9, CD63, and CD81 markers, in all preparations except for the extender. It is plausible that the extender contains vesicles derived from soy lecithin. In fact, when we characterized the miRNA content in all preparations, only a very small proportion of the total reads obtained from extender samples identified miRNAs, and expression of these was very different from other preparations.

Some of the most abundant miRNAs found in E (bta-miR-11980, bta-miR-11987, bta-miR-12057, bta-miR-1246, bta-miR-125b, bta-miR-181b, bta-miR-2340, bta-miR-2358, bta-miR-2478, bta-miR-2898, and bta-miR-345-3p), were also found significantly highly expressed in SPE or S samples compared to SP and are derived from extender addition. To the best of our knowledge these miRNAs have not previously been reported to be associated with sperm fertility. Finally, cryopreservation seems not to alter miRNAs composition. Changes in miRNA composition between sperm isolated from fresh ejaculate and after cryopreservation were previously observed in mice and human (36) and bull sperm (37). To the best of our knowledge seminal plasma alteration after cryopreservation was previously assessed exclusively by metabolomics studies, showing a strong difference between fresh seminal plasma and seminal plasma isolated from cryopreservation straws but without considering the effect of the extender addition (38).

## 5 Conclusion

In conclusion, the method used for EV isolation can influence the size and the quantity of spEVs isolated from preparations with and without extender. The commercial animal protein-free extender may contain phospholipid compounds, like soya lecithin, that form vesicle-like structures that can interfere with the correct spEV identification and characterization. The extender showed a lower miRNA cargo compared to sp-EVs preparation but several abundant miRNAs were present that were found significantly overexpressed in the S-EVs and SPE-EVs compared to spEVs. Although the EV profiles were influenced by the presence of the extender in S and SPE preparations, the miRNAs profile seems to be quite constant in the different preparations containing EVs. Nevertheless, in future studies involving the use of spEVs isolated from cryopreserved semen to evaluate potential markers of male fertility, it is advisable also to characterize the vesicles isolated from the extender used for straw preparation.

## Data availability statement

The data presented in this study are deposited in the NCBI Sequence Read Archive (SRA) (https://www.ncbi.nlm.nih.gov/sra) repository, accession number PRJNA1101695.

## Ethics statement

Ethical approval was not required for the study involving animals in accordance with the local legislation and institutional requirements because the study analyzed bull ejaculate collected from bovine semen production center.

## Author contributions

EC: Conceptualization, Data curation, Methodology, Writing – original draft. RF: Methodology, Writing – review & editing. BL: Data curation, Writing – review & editing. FT: Methodology, Writing – review & editing. GG: Methodology, Writing – review & editing. LP: Methodology, Writing – review & editing. AS: Supervision, Writing – review & editing. ALC: Writing – review & editing. FP: Conceptualization, Writing – review & editing. MC: Conceptualization, Funding acquisition, Writing – review & editing.

## Supplementary material

Supplementary Table S1 Progeny data and production, functionality, and type (PFT) index of bulls of proven fertility.

Supplementary Table S2 Characteristics of semen of proven bulls on pre-freeze and post-thaw samples.

Supplementary Table S3 SiMoA analysis reporting the average number of enzymes per bead (AEB), and the relative p-value for the comparison between the different preparations (SP-EVs, SPE-EVs, and S-EVs) and the extender (E-EVs) for UC and SEC isolation methods.

Supplementary Table S4 Sequencing statistics.

Supplementary Table S5 miRNA counts identified by MiRDeep2 and its modules.

Supplementary Table S6 Differentially expressed miRNA (DE-miRNAs) identified comparing different preparations. Value of Logarithmic Fold Change (logFC), Logarithmic Count Per Million (logCPM), *P*-Value, and False Discovery Rate (FDR) were reported.
